# Readiness of public schools before reopening during COVID-19 pandemic: School-based cross-sectional survey in southern Ethiopia

**DOI:** 10.1371/journal.pone.0293722

**Published:** 2023-10-31

**Authors:** Misganu Endriyas, Belete Woldemariam, Endashaw Shibru, Mamush Hussen, Bersabeh Bedru, Mathewos Moges, Mintesinot Melka, Fiseha Lemango, Male Mate, Tesfaye Lejiso, Biruk Gebremedhin, Alemu Tolcha, Biniam Shiferaw, Girma Wondimu, Tesfatsion Terefe, Sinafikish Ayele, Tebeje Misganaw, Teka Samuel, Temesgen Kelaye, Agegnehu Gebru, Amare Assefa, Wogene Getachew, Bereket Yalew, Dereje Geleta

**Affiliations:** 1 Health Research and Technology Transfer Directorate, SNNPR Health Bureau, Hawassa, Sidama, Ethiopia; 2 Disease Prevention and Health Promotion Directorate, SNNPR Health Bureau, Hawassa, Sidama, Ethiopia; 3 Head Office, SNNPR Health Bureau, Hawassa, Sidama, Ethiopia; 4 Public Health Institute Director Office, SNNPR Health Bureau, Hawassa, Sidama, Ethiopia; 5 College of Medicine and Health Science, Hawassa University, Hawassa, Sidama, Ethiopia; 6 Maternal, Child Health and Nutrition Directorate, SNNPR Health Bureau, Hawassa, Sidama, Ethiopia; 7 Medical Services Directorate, SNNPR Health Bureau, Hawassa, Sidama, Ethiopia; 8 Public Health Laboratory Directorate, SNNPR Health Bureau, Hawassa, Sidama, Ethiopia; 9 Public Health Emergency Management, Southwest Ethiopia Health Bureau, Tercha, Southwest Ethiopia, Ethiopia; 10 Health Research and Technology Transfer Directorate, Sidama Regional Health Bureau, Hawassa, Sidama, Ethiopia; 11 Transform Primary Health Care Project, Hawassa, Ethiopia; 12 Technical Assistant at EOC, SNNPR Health Bureau, Hawassa, Sidama, Ethiopia; Arba Minch University, ETHIOPIA

## Abstract

**Background:**

School closures in response to the COVID-19 impacted children’s education, protection, and wellbeing. After understanding these impacts and that children were not super spreaders, countries including Ethiopia decided to reopen schools with specified preconditions. But when deciding to reopen schools, the benefits and risks across education, public health and socio-economic factors have to be evaluated. However, there was information gap on status of schools as per preconditions. Hence, this study was designed to investigate status of schools in Southern Ethiopia.

**Methods:**

School based cross-sectional study was conducted in October 2020 in Southern Ethiopia. Sample of 430 schools were included. National school reopening guideline was used to develop checklist for assessment. Data was collected by public health experts at regional emergency operation center. Descriptive analysis was performed to summarize data.

**Results:**

A total of 430 schools were included. More than two thirds, 298 (69.3%), of schools were from rural areas while 132 (30.7%) were from urban settings. The general infection prevention and water, sanitation and hygiene (IPC-WASH) status of schools were poor and COVID-19 specific preparations were inadequate to meet national preconditions to reopen schools during the pandemic. Total score from 24 items observed ranged from 3 to 22 points with mean score of 11.75 (SD±4.02). No school scored 100% and only 41 (9.5%) scored above 75% while 216 (50.2%%) scored below half point that is 12 items.

**Conclusion:**

Both the basic and COVID-19 specific IPC-WASH status of schools were inadequate to implement national school reopening preconditions and general standards. Some of strategies planned to accommodate teaching process and preconditions maximized inequalities in education. Although COVID-19 impact lessened due to vaccination and other factors, it is rational to consider fulfilling water and basic sanitation facilities to schools to prevent communicable diseases of public health importance.

## Background

Coronavirus disease 2019 (COVID-19) is viral disease with main clinical symptoms of fever, dry cough, fatigue, myalgia, and dyspnea [1, 2]. Despite many efforts to control COVID-19, the disease continues causing huge social and economic impact and stress on the healthcare system [3, 4] and education system [5]. Presence of endemic illnesses such as malaria and influenza [6] and poor access to sanitation and lack of access to clean water made prevention of COVID-19 challenging in African settings [6, 7]. The recommended disease prevention methods during early introduction of disease were social distancing, wearing facemasks, handwashing, and quarantining contacts [8, 9]. School closure was also one of the key strategies to control spread of the virus [10–12].

In March 2020, the first case of COVID-19 was registered in Ethiopia and in response as part of the pandemic control, the above-mentioned measures, including school closure were applied. However, evidence suggested that school closures can reduce transmission of pandemic influenza if instituted early in outbreaks and are more likely to have the greatest effect if the virus has low transmissibility and if attack rates are higher in children than in adults [13]. In addition, it was noted that the school closures in response to the COVID-19 pandemic presented huge risks to children’s education, protection, and wellbeing, and maximized risk of violence, early pregnancy and more. In addition, evidences show the longer they are out of school, the less likely they are back to school [14]. Moreover, it also impacted life of teachers [10, 15].

Then after understanding these evidence that children were not super spreaders and pandemic impacts, countries decided to reopen schools with specified preconditions [16, 17] although there were fears that it may worsen health system unless controlled [18, 19]. It was recommended that when deciding to reopen schools, the benefits and risks across education, public health and socio-economic factors have to be evaluated [14]. So, the government of Ethiopia had set different preconditions before reopening schools that had taken infrastructure, waste management, sanitation, facemask, physical distancing, risk communication, management of suspected cases and others into account (See detail under variables in method section) [20].

Despite these preschool reopening preconditions, the status of schools in Southern Nations, Nationalities and People’s Region (SNNPR) of Ethiopia was unknown. So, this study was designed with the objective of evaluating the status of schools in SNNPR Ethiopia thereby to inform responsible authorities to take actions before school reopening and minimize the risk of disease transmission.

## Methods

### Study design and setting

School based cross-sectional study was carried out from October 15 to 25, 2020 in SNNPR. SNNPR was the third largest region representing nearly 15% population of the country. The region was administratively divided in to 17 zones and seven special woredas (districts). Woredas, sometimes called districts, are administrative structure of about 100,000 population and sub-divided into smallest administrative structure called *kebeles*. According to bureau of education, the region had 5,793 primary schools and 728 secondary schools. Currently, the region is divided in to three regions: South Ethiopia, Central Ethiopia, and Southwest Ethiopia regions.

### Sample size and sampling

Sample size for facility survey is usually determined by considering existing logistics and other relevant issues [21, 22]. Sample size was determined by using the following formula suggested for facility survey [21].

n=[zα2]2fqV2p

Where n = sample size, f = design effect, p = anticipated proportion of facilities with attribute of interest, q = 1-p, V^2^ = relative variance (square of the relative error) and Z is reliability coefficient at 95 percent level of confidence.

Assuming p (proportion of schools fulfilling COVID-19 prevention preconditions) 50% at 95% level of confidence, 15% relative error and design effect of 2.5, sample size was 427. Considering finite population correction and non-response rate of 10% (assuming closure of schools), final sample size was 441.

The estimated sample size was allocated to administrative structures proportionally based on number of schools obtained from the regional education bureau. But, at lower levels, schools were selected based on specific criteria. These were risk of the local areas based on community mobility, number of cases reported, population density and number of students per schools. Those schools with relatively higher rate of risks were included in the assessment.

### Data collection procedures

Data was collected by interviewing school principals, document review and observation of preconditions for school reopening. To minimize bias, data collection was done and monitored by public health experts who were members of regional emergency operation center (EOC), and items were verified by observation. The data collection tool (S1 Checklist) was adopted from checklist developed by ministry of health for school reopening. The adopted tool was reviewed by team of experts in EOC who were from bureau of education and health. The form was uploaded to Google form with skip patterns for data collection.

### Variables

We included the following variables in the assessment: establishment of committee to lead school reopening and its plan, assignment of IPC-WASH (Infection Prevention and Control and Water, Sanitation and Hygiene) supervisor, establishment of separate entry and exit gates, presence of isolation room and mini clinic (equipped with BP apparatus, infrared thermometer, bed and staff), risk communication materials, facemasks, sufficient class room to maintain social distance of one meter, adequate water (two litters per day per person), separate latrines for male (one hole for 40 students) and girls (one hole for 20 students) with handwashing facilities, sufficient handwashing facility with continuous water and soap supply at critical places such as at entrance and exit, sufficient waste management like incinerator (one per school) and waste pit (one per compound), at least one waste bins with cover/lid per class-room, and others [20].

### Data management

The collected data (S1 Data) was imported to SPSS version 25 for data management. The items observed were categorized to “yes” or available based on adequacy of national criteria or “no” if items were not available or not adequate as per national criteria. Descriptive statistics was performed to summarize data and results were presented using frequencies and percentages. The mean score difference between urban and rural was tested by independent t-test. To summarize the findings, items assessed were summed and the resulting composite was categorized into good (fulfilling 18 items or 75% of items), fair (fulfilling 50 to 74%) or poor (fulfilling less than 50% of items).

### Ethical considerations

Ethical clearance was obtained from IRB of SNNPR regional health bureau (Ref. £’6-19/7519). Informed verbal consent was approved and taken from school principals. Data collection form was set to end interview if consent was recorded as “no”. Support letter and messages to be aware of assessment were sent to schools by bureau of education. Although contact of person in charge of facility was collected for further follow-up like training, it was deleted from dataset and no personal identifier was used in analysis and report writing, and all collected data were kept confidential. Results of survey were disseminated to decision makers from administrative office, education, and health sectors (regional and zonal levels) immediately after completion of study and before school reopening.

## Results

### Background of schools

A total of 430 schools were included with response rate of 97.5%. Non-responses were due to closure of schools and unavailability of responsible person around. More than two thirds, 298 (69.3%), of schools were from rural areas while 132 (30.7%) were from urban settings. More than three fourths, 338 (78.6%), of schools were elementary schools while 92 (21.4%) were secondary schools.

### General infection prevention, water, and sanitation status of schools

**Fig 1:**
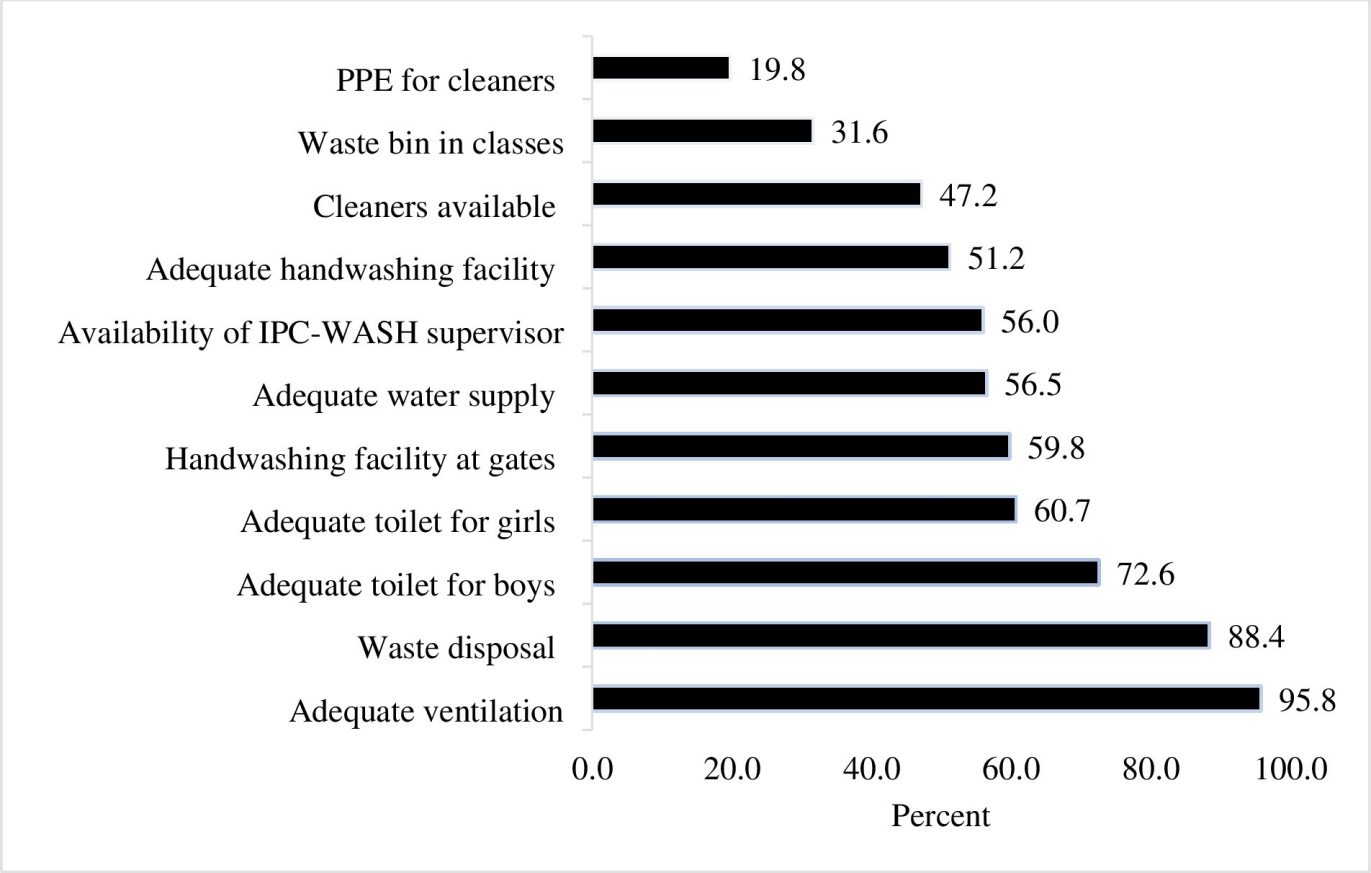
General infection prevention, water, and sanitation status of schools.

Most schools had adequate ventilation (95.8%) and waste disposal (88.6%). Adequacy of toilet for male students (one hole for 40 students) was achieved by 72.6% of schools while 60.7% had adequate toilet for girls (one hole for 20 students). Only less than three fifths (56.5%) of schools had adequate water supply (two liters per student per day) and only about half (51.2%) of schools prepared adequate handwashing facilities (one tap for 25 students). Only 47.2% of schools had cleaners and 19.8% schools had PPE for cleaners (Fig 1).

### COVID-19 specific infection prevention status of schools

**Fig 2:**
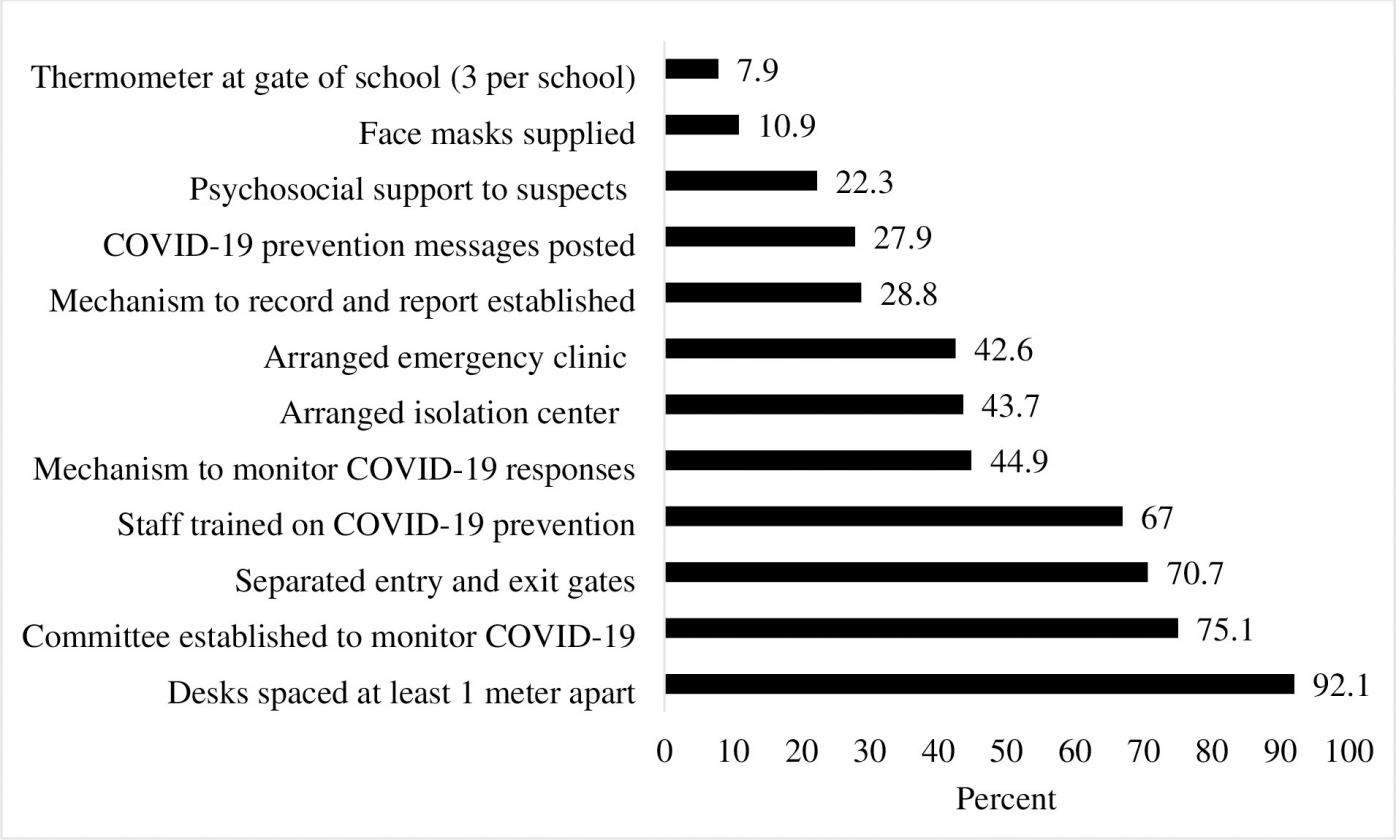
COVID-19 specific infection prevention status of schools.

Majority of schools (92.1%) arranged desks in class at one meter distance to maintain social distancing, 75.1% established committee to monitor COVID-19 responses and 70.7% separated entry and exit gates. Only 42.6% schools arranged clinics to provide emergency services and to link identified cases with health facilities while 188 (43.7%) arranged isolation center. As low as 10.9% of schools received facemask supplies for students and 7.9% schools had adequate thermometer (three per school) (Fig 2).

In summary, total score from 24 items observed ranged from 3 to 22 points and no school scored 100% and only 41 (9.5%) scored above 75% while 216 (50.2%%) scored below half point that is 12 items. The mean score was 11.75 (SD±4.02); 11.51 for urban and 11.86 for rural and there was no difference in mean scores (t_277.59_ = 0.88, p-0.38).

To compensate shortages of classrooms and implement preconditions set by government, schools planned extra shifts. That is, when desks were arranged one meter apart and number of students per class was reduced to maintain social distancing, number of students that schools can accommodate and number of regular students attending at schools were not balanced. So, some schools arranged extra shifts, up to four rounds (Table 1).

**Table 1:** Number of shifts schools planned to implement during COVID-19 pandemic.

Number of shifts planned to compensate preconditions	Frequency	Percent
One shift	16	3.7
Two shifts	225	52.4
Three shifts	182	42.3
Four shifts	7	1.6
Total	430	100

## Discussion

This study was done with aim of evaluating status of schools in SNNPR before school reopening during COVID-19 pandemic. Key findings include that the general IPC-WASH status of schools was poor, and COVID-19 specific preparations were inadequate to meet national preconditions to reopen schools during the pandemic. No school was perfect and very few scored above 75% points.

While evaluating the benefit of school reopening, assessing contextual public health and socio-economic factors in relation to the pandemic prevention and control are recommended [14], from which IPC-WASH status of schools is priority issue. The result of this study shows that the general IPC-WASH status of schools was poor as majority of items observed were not present in about half of schools. In cases when infrastructure is missing or in poor standard, it is challenging to maintain the quality of standards, especially during the emergencies. Previously, a study done in study setting also reported that two of the six schools assessed had medium level of handwashing facilities while four of them had poor status. In addition, the same study reported that only 22.3% students practice proper handwashing [23]. Such low access to hand hygiene was also noted in a study done in Nigeria [24]. IPC-WASH status is not only for the pandemic control but also has public health importance for prevention and control of many communicable diseases, especially in study setting where parasitic and bacterial infections are high and sanitation facilities are poor. Therefore, it is recommended that hand hygiene should be monitored rigorously at regular intervals, especially at school arrival, before drinking or eating, after using the toilet, after playing outdoors and like [17].

Face mask utilization is an important COVID-19 prevention method and highly reduces the risk of disease propagation, especially if both cases and at-risk people properly use it [25]. Although children are less likely to show symptoms of COVID-19 or their illness is usually mild, the use of facemask especially by high-schoolers could decrease the spread to the virus [26–28]. But, during early introduction of the pandemic, in study setting, facemask was not known by the community and even majority of healthcare workers had limited access to it. In addition to its unavailability to the public, its cost was not affordable to low-income households. So, government and partners were distributing facemasks to schools before reopening. However, as much as 89.1% of schools had no facemasks for students.

In study setting, majority of students are from rural areas where there is limited access to provide technology-based education like through internet, TV, radio etc., or even if it was attempted, it would have maximized inequalities between students. So, face-to-face education was found critical, and schools had modified their usual shifting schedule to accommodate students and maintain social distancing. In most cases of study settings, schools have two shifts (morning and afternoon). But to accommodate catchment students, some schools planned up to four shifts, probably about two hours education. On the top of existing inequalities in accessing quality education and differences in capacity of schools to prevent the pandemic, it is possible to imagine the inadequacy of education provided within this short time that maximizes the existing inequalities.

Studies [29–32] have shown the impact of COVID-19 on education in terms of performances. But our results show the impact of COVID-19 in terms of school readiness to provide education while preventing the disease in resource limited setting. Even though we could reach to large number of facilities in high-risk areas while there was high level of fear and risk of traveling associated with COVID-19, our study was limited in addressing private schools, and also assessing other possible risks factors like available mode of transportation for students. In addition, the study was limited to report the actual infection prevention practice because schools were closed at the time of data collection.

## Conclusion

Both the basic and COVID-19 specific IPC-WASH status of schools were inadequate to implement national school preopening preconditions and basic standards. In addition to inequalities in risk of infection, some of strategies to accommodate teaching process and preconditions maximized inequalities in education. Although COVID-19 impact is lessened now due to vaccination and other factors, it is rational to consider fulfilling water and basic sanitation facilities to schools to prevent communicable diseases of public health importance.

## Supporting information

S1 Checklist Checklist used to collect data. (DOCX)

S1 Data Data in SPSS sav file type. (SAV)

## Lists of abbreviations

EOC: Emergency Operation Center
IPC: Infection Prevention and Control
PPE: Personal Protective Equipment
SNNPR: Southern Nations Nationalities and People’s Region
WASH: Water, Sanitation and Hygiene
