# Supplementary material for: Readiness of public schools before reopening during COVID-19 pandemic: School-based cross-sectional survey in southern Ethiopia
Source: PLoS One. 2023 Oct 31;18(10):e0293722. doi: 10.1371/journal.pone.0293722 (PMC10617685; doi:10.1371/journal.pone.0293722)
Supplement: S1 Checklist — (DOCX) [file pone.0293722.s001.docx]

**Checklist for assessment of school readiness before school reopening during COVID-19 pandemic**

Date of assessment: ________________

Zone/Special wereda: _______________

Wereda: __________________________

Name of school: ____________________

School type: Primary____ Secondary _____

School place: Town ______ Rural_________

Name of school principal/supervisor/contact person: __________________Phone: ___________

Instruction: Find appropriate person for interview, preferably the principal. Explain the purpose and objective and get consent. Ask each of the following items and verify by observation. If an item is available as per national standard, give 1 and if an item is unavailable or inadequate, say no (0). For numeric responses and any additional comments, use comment section.

| S.N | Questions | Yes (1) | No (0) | Comment |
| --- | --- | --- | --- | --- |
| General school setup | | | | |
|  | Number of teachers/staffs |  |  |  |
|  | Number of male students |  |  |  |
|  | Number of female students |  |  |  |
|  | Is there separate door for entry and exit? |  |  |  |
|  | Presence of Staff clinic |  |  |  |
|  | Presence of isolation center |  |  |  |
|  | How many shifts are planned |  |  |  |
| Theme 1 Organizational Management | | | | |
|  | Availability of IPC-WASH committee |  |  |  |
|  | Availability of COVID prevention and response plan which is led by school administration (observe the plan considering COVID 19) |  |  |  |
|  | Availability of IPC-WASH supervisor or focal |  |  |  |
|  | Availability of water (5 liter per student) |  |  |  |
|  | Availability of functional hand washing facilities at different corners of the school (1 tap for 25 students) |  |  |  |
|  | Availability of hand washing facility or hand rub at the gates of the school and the classes (Entry, exit, classroom blocks) |  |  |  |
|  | Presence of posted COVID-19 prevention messages and risk communication with Hotline phone number 8335 (observe the messages) |  |  |  |
|  | Is there adequate face mask ready for students and staffs |  |  |  |
|  | Presence of screening at entrance gate (Thermo-scan, at least 3/school) |  |  |  |
|  | Is there functional toilet for male students? (1 hole for 50 males) |  |  |  |
|  | Is there functional toilet for female students? (1 hole for 25 girls) |  |  |  |
|  | Is there functional hand washing facility adjacent to toilets. |  |  |  |
|  | Is there functional separate toilet with hand washing facilities for staffs? (1 hole for male and 1 for female staffs) |  |  |  |
|  | Presence of functional windows for room ventilation? |  |  |  |
|  | Staff trained/oriented on COVID-19 prevention |  |  |  |
|  | Is there any mechanism to follow and monitor services with regards to COVID-19 prevention daily bases (observe the plan) |  |  |  |
|  | Is there recording and reporting mechanism on COVID 19 prevention and response activity (observe formats) |  |  |  |
|  | Are the classroom desks spaced at least 1meter apart to keep physical distance in between students (20-25 students per class/room) (observe number of desks in the room and registered students) |  |  |  |
|  | Presence of monitoring for school community to keep physical distance (staff lounge, distance between blackboard and desk, flag ceremony) |  |  |  |
|  | Presence of functional staff clinic or linkage to nearby health facilities |  |  |  |
|  | Is there psychosocial support for the identified cases? |  |  |  |
| Theme 2 Cleaning & Waste management | | | | |
|  | Does the school have cleaner? |  |  |  |
|  | Are there mechanisms to clean & swab with disinfectant for repeatedly touched areas and surfaces (observe sanitizer and disinfectant) |  |  |  |
|  | Is there proper PPE for cleaner? (Utility glove, bouts, gown, face mask) |  |  |  |
|  | Do cleaners utilize appropriate PPE during cleaning? |  |  |  |
|  | Waste bins are prepared for classes and compound (1 per classroom) |  |  |  |
|  | Availability of final waste disposal mechanism (incinerator, pit) (1 per compound) |  |  |  |

Name of assessors - ---------------------------- Signature ---------------
